# Changes in gene expression between a soybean F_1_ hybrid and its parents are associated with agronomically valuable traits

**DOI:** 10.1371/journal.pone.0177225

**Published:** 2017-05-11

**Authors:** Earl Taliercio, David Eickholt, Rakin Rouf, Thomas Carter

**Affiliations:** 1 USDA-ARS, Raleigh, North Carolina United States of America; 2 Crop and Soil Science Department, North Carolina State University, Raleigh, NC, United States of America; New Mexico State University, UNITED STATES

## Abstract

Soybean [*Glycine max* (L.) Merr.] genetic diversity is limited because domesticated soybean has undergone multiple genetic bottlenecks. Its progenitor, the wild soybean [*Glycine soja* Siebold & Zucc], has not undergone the same intense selection and is much more genetically diverse than domesticated soybean. However, the agronomic importance of diversity in wild soybean is unclear, and its weedy nature makes assessment difficult. To address this issue, we chose for study a highly selected, adapted F_4_-derived progeny of wild soybean, NMS4-44-329. This breeding line is derived from the hybridization between *G*. *max* cultivar N7103 and *G*. *soja* PI 366122. Agronomic comparisons were made among N7103, NMS4-44-329 and their F_1_ and F_2_ progeny in replicated yield trials at two North Carolina locations. Significant F_1_ mid-parent heterosis was observed at each location for seed yield (189 and 223 kgha^-1^, P<0.05 and P<0.10, respectively), seed protein content (1.1g/100g, P<0.01) and protein production per hectare (101 and 100 kgha^-1^, P<0.01 and P<0.06, respectively). Increased yield, seed protein content and protein production per hectare in the hybrids suggested that wild soybean has the potential to improve agronomic traits in applied breeding. Comparisons of differentially-expressed genes in the hybrid vs. parents identified genes associated with N metabolism. Non-additive changes in gene expression in the hybrids relative to the parents could reasonably explain the improved protein levels in the F_1_ hybrids. Changes in gene expression were influenced by environmental effects; however, allele specific bias in the hybrids were well correlated between environments. We propose that changes in gene expression, both additive and non-additive, and changes in allele specific expression bias may explain agronomic traits, and be valuable tools for plant breeders in the assessment of breeding populations.

## Introduction

Plant breeding consists primarily of creating genetic and phenotypic variation by hybridizing desirable contrasting parents, and selecting recombinant progeny which out-perform the parents. Finding parents that are both desirable and genetically diverse among elite cultivars can be challenging for a plant breeder in a crop that has undergone multiple genetic bottlenecks, such as soybean [1,2]. Molecular tools have become more common in plant breeding in recent years to address this problem, because they are extremely useful for identifying and tracking genetic diversity. For example, researchers used a 50K SNP platform to characterize the diversity inherent in domesticated soybean [*Glycine max* (L.) Merr.] and its wild progenitor [*Glycine soja* Siebold & Zucc.] [3,4]. This information can be used by plant breeders to identify and select parents which not only have desirable traits, but are also genetically diverse. In many crops, the chance of developing agronomically desirable progeny which are superior to the parents is increased by focusing on the genetic distance between parents. In corn, increased genetic distance is thought to enhance heterosis, which is defined as the improved performance of the F_1_ hybrid over either parent [5]. However, in maize, the association between genetic distance and heterosis is not always sufficiently great to have predictive value [6,7]. In soybean, because of the self-pollinating mode of reproduction, heterosis and hybrid vigor do not play a significant role in current production, although the existence of heterosis has been reported [8–10]. When F_1_ heterosis is present for quantitative traits such as seed yield, it may indicate that the parents contrast sufficiently so that the diversity between the parents can be captured in a transgressive recombinant progeny. The use of heterosis as a screening tool to select desirable parental combinations has been proposed by Brownie and Burton (1993) in soybean.

Genetic markers have been widely adapted for plant breeding in many crops, including soybean. Another tool that is just beginning to be utilized by plant breeders is genotyping by sequencing (GBS) [11]. Next generation sequencing allows the generation of sequence data from a variety of plant samples. Analyses of these data requires some specialized knowledge of handling large data sets, infrastructure to handle the data sets, and sufficient funds to sequence the number of samples required for statistical power. Nonproprietary programs, well supported web interfaces, and cloud computing address the first two requirements for dealing with sequencing data. The third requirement, the cost of sequencing, is nearing the threshold of being an affordable alternative to marker based analyses. Both deep sequencing of genomes and sequencing of expressed genes (RNAseq) are becoming more affordable and can be used in GBS.

Although the genetic diversity in the USDA domesticated soybean germplasm collection is substantial and agronomically useful, the genetically diverse progenitor species, wild soybean has received little attention by applied breeders. Thus, the true agronomic importance of diversity preserved in the USDA wild soybean collection is unclear with respect to plant breeding [12]. A central problem in the study of wild soybean is that its viny nature makes agronomic assessment difficult. Not only is the wild soybean itself completely unadapted to row crop production, but so are most of the progeny derived from crosses between *G*. *soja* and *G*. *max*. To circumvent the problem of poor plant architecture in the wild soybean and, thus, facilitate assessment, we chose to study a rare, highly-selected, adapted and upright F_4_-derived progeny of wild soybean, NMS4-44-329. NMS4-44-329 was developed in the USDA soybean breeding program at Raleigh, NC [13] and is derived from the hybridization of *G*. *max* cultivar N7103 [14] and *G*. *soja* PI 366122. NMS4-44-329 was hybridized (backcrossed) with its domesticated parent N7103 to produce 3900 F_1_ hybrid seeds and 23 F_2_ populations.

In this study, we evaluate the agronomic impact of the wild grandparent on yield and seed composition of the F_1_ and F_2_ progeny from the cross between N7103 and NMS4-44-329. We also correlate detailed gene expression data from RNAseq analyses with agronomic data from the two soybean parents with their F_1_ progeny in replicated yield trials in two environments. The parents, N7103 and NMS4-44-329, have similar agronomic performance, but differ substantially in sequence diversity as a consequence of the wild soybean in the pedigree of NMS4-44-329. The availability of NMS4-44-329 allowed us to correlate agronomic traits that contrast between the F_1_ hybrids and the parents with differentially expressed genes in the F_1_ hybrids. We also took advantage of the diversity contributed by the wild grandparent (PI 366122) to identify alleles that had biased expression (>73% one allele) in the F_1_ hybrids. Finally, we used analyses of the genes that were differentially expressed in the F_1_ hybrids to gain insight into the molecular basis of agronomic traits and propose that aspects of gene expression and allele specific expression may be useful tools for soybean breeding.

## Materials and methods

### Parental pedigree

NMS4-44-329 is a F_4_-derived breeding line from the hybridization of USDA cultivar N7103 and the USDA *G*. *soja* accession PI 366122 [14]. N7103 has 100 percent *G*. *max* pedigree and is a F_4_-derived cultivar from the hybridization of USDA breeding line NTCPR90-143 and USDA cultivar ‘Pearl’ (PI 583367) [15]. Both breeding line and cultivar are maturity group VII materials adapted to the Southeastern USA. NMS4-44-329 is a rare adapted and upright breeding line developed through extensive selection among F_3_ plants followed by pedigree selection and yield trials.

### Production of seed for the experiment

In 2013 and 2014, hybridizations were made between N7103 as the female parent and NMS4-44-329 as the male parent. Approximately 3,900 F_1_ seed were generated at the Central Crops Research Station (CCRS) in Clayton NC. The CCRS is a part of the North Carolina Department of Agriculture and the North Carolina State University Research system. In the winter of 2014-2015, 23 F_1_ plants were grown and individually harvested at the USDA Tropical Agriculture Research Station (winter nursery) at Isabella, Puerto Rico, to produce F_1:2_ seed. Self-pollinations were removed from the winter nursery prior to harvest, by discarding plants with green hypocotyl color at the seedling stage or white flowers at mid-bloom. Purple flower color and purple hypocotyl are dominant to white flower and green hypocotyl color and pleiotropic. Because the female N7103 has white flowers and green hypocotyls and male breeding line NMS4-44-329 has purple flowers and purple hypocotyls, F_1_ plants are expected to have purple color [16]. Parental seed stock sources for the experiment, as well as seed for the four maturity checks (USDA NC-Roy, NCC06-1090, USDA N7003CN and USDA N8002), were produced in 2014 in North Carolina.

### Agronomic evaluation

An experiment was developed to collect agronomic data in standard-size breeder plots for seed yield, seed protein and oil content, plant maturity, plant erectness, plant height and 100-seed weight. The experiment was conducted at two locations in 2015 (Caswell Research Station (CRS), Kinston, NC; CCRS, Clayton, NC), and utilized a randomized complete block design (RCBD), with five blocks per location. The CRS is a part of the North Carolina Department of Agriculture and the North Carolina State University Research system. Each block included one F_1_ entry and three F_2_ entries. Six entries of NMS4-44-329 and N7103 were also included. In addition, each block included the following maturity checks: USDA NC-Roy, NCC06-1090, USDA N7003CN and USDA N8002 [17–19]. All maturity checks were replicated two times per block, except for NCC06-1090, which was replicated three times per block. Repeated entries were included within a block to increase the testing precision for heterosis analysis. Over the two locations, a total of ten replications were grown for the F_1_, 30 for the F_2_ and 60 for the parents, NMS4-44-329 and N7103. We have included the raw field data as S1 Table.

### Field evaluation

The experiment was planted at Clayton on 28 May 2015, and at Kinston on 13 June 2015, in North Carolina under authority of North Carolina State University and North Carolina Department of Agriculture. Each experimental unit consisted of 3-rows planted with an inter-row spacing of 0.97 m, and a planting length of approximately 3.35 m. Approximately two weeks after planting, putative F_1_ plants were examined and those which did not have the expected purple hypocotyl color were treated as contaminants and removed. Approximately one percent of seedlings were removed from the hybrid plots using this protocol. At maturity, plots were end trimmed to a uniform length of 2.74 m. All agronomic data for the study were collected only on the center bordered row.

### Seed composition analysis

Seed protein and oil content measurements were taken using a Perten DA 7250 near-infrared (NIR) spectrometer (Perten Instruments, Springfield, IL). Equations used for the NIR analysis were provided by Perten for the year 2015. Concentrations were reported in a zero moisture basis.

### Statistical analysis

Statistical analyses of all agronomic and morphological data were performed using the Statistical Analysis System 9.4 (SAS Institute, Cary). To identify potential outliers in the agronomic data set, an initial set of analyses of variance was performed for each location separately, using Proc GLM. Genotype was treated as a fixed effect and block as a random effect. Subsequently, a trend analysis of residuals was added using fourth-degree polynomials for the row and column positions of plots, which were treated as fixed effects [20]. Externally studentized residuals were calculated for each plot and trait, and observations were dropped if associated with a studentized residual greater than three. Two observations were dropped from the data set using this approach. The reduced data set was then subjected to an additional analysis of variance combined with trend analysis, again employing row and position effects as covariates. Sequential sums of squares (Type 1) output were evaluated and row and column covariates which were not significant (P < 0.05) were dropped, starting with the highest order covariate. If a higher order covariate was significant, but a lower order covariate was not, the lower order covariate was left in the model. A final model retaining significant (P < 0.05) row and position effects was used in all further analysis of agronomic traits.

After outlier removal and trend analysis, isotropic and two-dimensional anisotropic exponential covariance structures were examined for the dependent variable yield using Proc Mixed. A final model was selected based on likelihood ratio testing (LRT). For the CRS location, the standard independent and identical error distribution (IID) variance-covariance structure was the best fit. For the CCRS location, a two-dimensional geometrically anisotropic variance-covariance structure was the best fit. At a given location, the best-fitting type of variance-covariance structure for yield was then applied to other agronomic trait analyses, since yield was assumed to best capture true field variation. After a final model was selected, single degree of freedom linear contrasts between genotypes were performed using the contrast statement. In addition to separate location analyses, pooled analyses over locations were also performed for each trait using Proc Mixed. Genotype and trend covariates nested within location served as fixed effects, whereas location, genotype x location (GxE) and block nested within location served as random effects. Three different residual covariance structures were evaluated in the analyses over locations: 1) IID, 2) Different variance for each location, IID within location and 3) Separate variance for each location, two-dimensional anisotropic exponential variance within location. Using LRT, the best model had a separate residual variance for each location, with an IID structure within locations. This same model was applied to all other agronomic traits. After final model selection, linear contrasts were again performed using contrast statements. We observed that two samples, representing an F_1_ hybrid in Clayton and an N7103 parent in Kinston, were outliers for both the agronomic data and subsequent analyses of gene expression.

### RNA isolation and analysis

For RNA analysis, the most recently fully expanded trifiolate was harvested from five plants of the F_1_ and parental genotypes in each of 4 blocks at each location, just prior to flowering. Although parental lines were replicated six times per block, only one parental replicate per block was used. Leaves were harvested from Kinston on 15 July 2015 at 9 AM and from Clayton on 10 July 2015 at 9 AM. RNA was isolated using the Qiagen plant RNeasy isolation kit (Qiagen, Redwood City, CA) with on-column DNAse digestion. RNA quality was validated in a 2100 Bioanalyzer (Agilent Technologies, Santa Clara CA). TruSeq RNA libraries from four replicates of each parent and F_1_ hybrid were sequenced by HiSeq (Illumina, San Diego, CA). After removing primer sequences and low quality sequences with a Phred score less than 34, between 11 million and 32 million high quality sequences remained. Table 1 shows the number of high quality sequences for each sample. The high quality RNA sequences were aligned to the reference genome (Wm82.a2) using TopHat and the number of sequences that align to the reference genome are shown in Table 1 [21]. Gene expression was analyzed using the Cuffdiff 2 program [21]. Differential expression files and raw sequence files are available from the Gene Expression Omnibus (GSE86608).

**Table 1:** 

sample	Description	total	aligned	percent
Sample 31	F_1_ hybrid rep 1 Clayton	28707416	26315343	91.67
Sample 32	F_1_ hybrid rep 2 Clayton	21502547	20064518	93.31
Sample 33	F_1_ hybrid rep 3 Clayton	12734640	11737193	92.17
Sample 35	F_1_ hybrid rep 4 Clayton	23041971	21426802	92.99
Sample 38	NMS4-44-329 rep 2 Clayton	21415384	20018568	93.48
Sample 39	NMS4-44-329 rep 3 Clayton	20866477	19570654	93.79
Sample 40	NMS4-44-329 rep 4 Clayton	27198597	25808651	94.89
Sample 41	N7103 rep 1 Clayton	29895313	27619619	92.39
Sample 42	N7103 rep 2 Clayton	35857178	32714854	91.24
Sample 43	N7103 rep 3 Clayton	34188660	31970599	93.51
Sample 45	N7103 rep 4 Clayton	24153514	22823047	94.49
Sample 46	F_1_ hybrid rep 1 Kinston	30967667	29591485	95.56
Sample 48	F_1_ hybrid rep 2 Kinston	27346296	25826404	99.79
Sample 49	F_1_ hybrid rep 3 Kinston	23069040	20029815	86.83
Sample 50	F_1_ hybrid rep 4 Kinston	25579134	23746216	92.83
Sample 52	NMS4-44-329 rep 1 Kinston	33056837	24520694	74.18
Sample 53	NMS4-44-329 rep 2 Kinston	27409038	25426810	92.77
Sample 54	NMS4-44-329 rep 3 Kinston	24371873	22287564	91.45
Sample 55	NMS4-44-329 rep 4 Kinston	28604242	26099606	91.24
Sample 56	N7103 rep 1 Kinston	20250602	18769834	92.69
Sample 58	N7103 rep 2 Kinston	30324958	28289439	93.29
Sample 60	N7103 rep 4 Kinston	30901625	28381274	91.84

Information for the sequence and alignment information including, the sample names, a description of the samples, the total number of sequences after trimming, the number of sequences that aligned to the reference genome and the percent of sequences that aligned to the reference genome.

The alignments to the reference sequence were also used to generate variant call files using mpileup. Sequences 2 bp either side of splice junctions were omitted because they have a higher chance of misalignment. Parent specific SNPs were identified that were present in all parental plots sequenced at CRS and their presence in all appropriate parental sequences from CCRS was confirmed. SNPs unique to each parent were identified to generate a list of high quality SNPs specific to each parent. Next, reads that over-lapped these parent-specific SNPs were identified in each F_1_ hybrid sequenced and the read depths (number of reads) were recorded from each F_1_ sample sequenced. The F_1_ hybrids contain one copy of each parental SNP and there was considered to be an allele bias if one allele accounted for more than 73% of the reads consistently across a location. The gene identity and the impact of the variants were determined using SNPEFF [22].

Singular Enrichment Analysis (SEA) of the gene ontologies (GO) was performed at AgriGO using the soybean reference genome Williams 82 [23]. The reference gene list used for SEA contained all of the genes expressed in the leaf tissue.

## Results

### Agronomic comparison

Agronomic data were evaluated for both parents, the F_1_ hybrid, F_2_ progeny and the maturity checks. Results from Kinston and Clayton showed that the F_1_ hybrid yielded, on average, 189 and 223 kg ha^-1^ more than the mean of the parents (mid-parent value) (P = 0.05 and 0.10, respectively) (Tables 2 and 3). The combined data over locations revealed an increase in yield in the F_1_ hybrids over the mid-parent of 182 kg ha^-1^ (P = 0.15). The F_2_ yields were lower relative to the mid-parent value in both environments. Increases in seed weight of 0.6g (P < 0.01), 0.9g (P< 0.01) and 0.7g (P = 0.11) were observed in the F_1_ hybrid relative to the mid-parent in Kinston, Clayton and over both locations, respectively. Maturity Date was significantly different (P < 0.05) in the F_1_ hybrid compared to the mid-parent at one location, but the numerical difference was only 1 day. The higher-yielding Clayton environment was associated with taller plants and reduced lodging, but this association was not observed in Kinston.

**Table 2:** Agronomic data.

	Kinston (CRS)							
	Yield	Protein	Harvestable Protein	Oil	Maturity	Lodging	Height	100-Seed Weight
	(kg ha^-1^)	(g 100g^-1^)	(kg ha^-1^)	(g 100g^-1^)	(Oct.1 = 1)	(1-5)	(cm)	(g)
F1	2204	46.0	941	20.3	38	3.3	66	10.1
F2	2045	46.1	873	20.1	38	3.4	65	9.7
NMS4-44-329	1934	44.1	791	20.6	38	4.1	57	10.7
N7103	2096	45.7	889	20.2	36	2.5	81	8.2
NC Roy	2606	44.4	1075	21.5	32	3.3	55	13.4
NCC06-1090	2555	40.7	964	23.7	35	2.7	70	16.2
N7003 CN	2163	42.7	857	23.0	36	2.9	74	15.7
N05-7432 IB	2443	45.0	1020	20.9	43	3.3	58	13.8
	Clayton (CCRS)							
	Yield	Protein	Harvestable Protein	Oil	Maturity	Lodging	Height	100-Seed Weight
	(kg ha^-1)^	(g 100g^-1^)	(kg ha^-1^)	(g 100g^-1^)	(Oct.1 = 1)	(1-5)	(cm)	(g)
F1	2618	44.1	1057	19.7	32	2.3	85	10.3
F2	2529	44.1	1053	19.8	33	2.3	84	9.6
NMS4-44-329	2479	42.2	971	20.5	31	3.1	75	10.9
N7103	2311	43.7	943	19.8	31	1.6	76	7.8
NC Roy	2870	43.1	1136	21.3	26	1.6	80	14.1
NCC06-1090	2393	40.8	912	23.8	24	1.5	74	17.9
N7003 CN	2726	40.5	1024	22.3	30	1.7	82	15.7
N05-7432 IB	2688	42.1	1021	20.8	34	2.5	78	14.0
	Both Locations							
	Yield	Protein	Harvestable Protein	Oil	Maturity	Lodging	Height	100-Seed Weight
	(kg ha^-1)^	(g 100g^-1^)	(kg ha^-1^)	(g 100g^-1^)	(Oct.1 = 1)	(1-5)	(cm)	(g)
F1	2301	45.0	960	20.1	35	2.7	76	10.1
F2	2180	45.1	910	20.0	35	2.8	75	9.6
NMS4-44-329	2118	43.1	843	20.6	35	3.6	66	10.8
N7103	2122	44.7	881	20.0	33	2.0	78	8.0
NC Roy	2647	43.7	1072	21.4	29	2.5	68	13.7
NCC06-1090	2428	40.8	917	23.8	30	2.1	72	17.0
N7003 CN	2348	41.5	899	22.7	33	2.3	77	15.6
N05-7432 IB	2439	43.6	989	20.9	39	2.8	68	13.8

Agronomic comparison of the parents, progeny and maturity checks for soybean grown at Clayton and Kinston, NC in replicated trials in 2015. Lodging is rated 1 (erect) to 5 (prostrate).

**Table 3:** Contrast table for agronomic data.

	Kinston (CRS)							
	Yield	Protein	Harvestable Protein	Oil	Maturity	Lodging	Height	100-Seed Weight
	(kg ha^-1^)	(g 100g^-1^)	(kg ha^-1^)	(g 100g^-1^)	(Oct.1 = 1)	(1-5)	(cm)	(g)
F1 vs.	190	1.1	101	-0.1	1	0	-3	0.6
Mid-parent	[0.05]	[0.01]	[0.02]	[0.58]	[0.04]	[0.74]	[0.40]	[0.01]
F2 vs.	31	1.1	33	-0.2	1	0.1	-4	0.2
Mid-parent	[0.33]	[0.01]	[0.13]	[0.01]	[0.01]	[0.31]	[0.06]	[0.03]
	Clayton (CCRS)							
	Yield	Protein	Harvestable Protein	Oil	Maturity	Lodging	Height	100-Seed Weight
	(kg ha^-1^)	(g 100g^-1^)	(kg ha^-1^)	(g 100g^-1^)	(Oct.1 = 1)	(1-5)	(cm)	(g)
F1 vs.	223	1.1	100	-0.4	1	-0.1	10	0.9
Mid-parent	[0.10]	[0.01]	[0.06]	[0.06]	[0.10]	[0.50]	[0.01]	[0.01]
F2 vs.	134	1.2	96	-0.3	2	0	9	0.2
Mid-parent	[0.10]	[0.01]	[0.01]	[0.02]	[0.01]	[0.61]	[0.01]	[0.23]
	Both Locations							
	Yield	Protein	Harvestable Protein	Oil	Maturity	Lodging	Height	100-Seed Weight
	(kg ha^-1^)	(g 100g^-1^)	(kg ha^-1^)	(g 100g^-1^)	(Oct.1 = 1)	(1-5)	(cm)	(g)
F1 vs.	182	1.1	99	-0.2	1	-0.1	4	0.7
Mid-parent	[0.15]	[0.10]	[0.07]	[0.31]	[0.48]	[0.53]	[0.60]	[0.11]
F2 vs.	62	1.2	48	-0.3	1	0	3	0.2
Mid-parent	[0.34]	[0.08]	[0.19]	[0.14]	[0.24]	[0.95]	[0.67]	[0.57]

Agronomic contrasts between the parents and their progeny for soybean grown at Clayton and Kinston, NC in replicated trials in 2015. Differences are from single degree of freedom contrasts and their associated P-values are in square brackets. Yield and harvestable protein P-values are from one-sided hypothesis testing. P = 0.01 represent values of 0.01 or less.

Analysis of seed composition data revealed that the F_1_ hybrid had significantly (P < 0.01) greater seed protein content than the mid-parent value at each location (1.1 g/100 g sample on a zero moisture basis, Tables 2 and 3). At both Kinston and Clayton, the F_1_ hybrids had significantly (P < 0.01) higher seed protein content than NMS4-44-329. Also, in contrast to the yield data, the elevated protein levels observed in the F_1_ hybrids persisted in the F_2_ progeny. Protein increased in the F_2_ progeny relative to the parents when analyzed by location, and over locations. There was no consistent significant difference in oil levels between the F_1_ hybrids and the mid-parent values across environments.

Seed protein production per hectare was also greater for the F_1_ hybrid than the mid-parent value at Kinston and Clayton (101 and 100 kgha^-1^, respectively, P≤0.02, P≤0.06). The seed protein production of the F_1_ was also numerically superior to each parent at each location and significantly (P<0.05) greater than that of the mid-parent value (P<0.06) averaged over locations (Table 3).

### Gene expression

We next determined if differences in gene expression could explain improved yield and seed protein levels observed in the F_1_ hybrids in field experiments. Table 4 shows the total number of genes expressed, as well as those genes differentially regulated (≥ 2 fold change in expression) between the parents and the hybrids. Over 33,000 genes were expressed at both locations. At Kinston, a total of 326 (0.96%) or 321 (0.95%) genes were differentially regulated in the F_1_ relative to N7103 or NMS4-44-329, respectively. At Clayton, a total of 452 (1.29%) or 320 (0.91%) of genes were differentially regulated in the F_1_ relative to N7103 or NMS4-44-329, respectively. When considering all genes differentially regulated in either parent relative to the hybrids, the correlation between differentially expressed genes between locations was low (R^2^ < 0.39). However, 95 genes differentially expressed between N7103 and the F_1_ hybrids at both locations were well correlated (R^2^ = 0.72). Ninety-four genes were differentially expressed in the same direction in the F_1_ hybrids relative to the NMS4-44-329 parent in both locations and with highly correlated expression (R^2^ = 0.91). This subset represents genes with genotype-specific expression that, at least in Kinston and Clayton, were not sensitive to environmental effects.

**Table 4:** Gene counts from gene expression study.

	Total Genes	F_1_>N7103	F_1_<N7103	F_1_>NMS4	F_1_<NMS4	F_1_> both	F_1_<both
Kinston	33677	219	107	161	160	25	6
Clayton	35026	203	249	156	164	20	31

Total counts of genes sequences from all samples harvested at Kinston or Clayton (total genes). Counts of transcripts in the F_1_ hybrids that: were greater than one parent (>NMS4-44-329 or >N7103); were less than one parent (<NMS4-44-329 or <N7103); were greater than both parents (>both); were less than both parents (<both). Genes that are greater than or less than both parents are the “non-additive” category.

SEA uses ontologies of genes altered in expression in the F_1_ hybrid relative to each parent to gain insight into potential physiological differences between the parents and their hybrid at each location. Analyses of the genes altered in expression in the F_1_ hybrids relative to the parents in both locations did not show any enrichment in GO categories. Table 5 shows a selected list of relevant enriched ontologies based on comparisons of the F_1_ hybrid with individual parents where enrichments were observed. No clear pattern of enrichment emerged for some ontologies. For example, categories related to oxidation-reduction were enriched in both locations among genes that are both up-regulated and down-regulated in the F_1_ hybrids relative to the parents. The category of “pectinesterase” was enriched among genes with elevated levels of expression in the parents in Kinston and may be related to the “cell wall” category with a similar pattern of expression. Perhaps the most interesting observation was that genes associated with nitrogen metabolism were enriched in expression in the N7103 parent relative to the F_1_ in Clayton where we saw a trend toward increasing seed protein. The subset of genes with genotype-specific expression in the hybrid compared to N7103 were enriched in genes associated with “amine metabolic process” (GO:0009308, P = 0.0004). However, the enriched ontologies associated with N metabolism were not significant among genes associated with the other parent even though the F_1_ hybrids have substantially more protein that NMS4-44-329.

**Table 5:** Enriched ontologies.

Location	Expression	GO term	Description	p-value
Kinston	F_1_>N7103	NONE		
Kinston	F_1_<N7103	GO:0055114	oxidation reduction	0.000038
Kinston	F_1_<N7103	GO:0045449	regulation of transcription	0.0022
Kinston	F_1_<N7103	GO:0019219	regulation of nucleobase	0.0024
Kinston	F_1_<N7103	GO:0051171	regulation of nitrogen compound metabolic process	0.0024
Kinston	F_1_<N7103	GO:0043531	ADP binding	0.000055
Kinston	F_1_>NMS4-44-329	NONE		
Kinston	F_1_<NMS4-44-329	GO:0055114	oxidation reduction	0.00000068
Kinston	F_1_<> both	GO:0043167	ion binding	0.017
Clayton	F_1_>N7103	GO:0009308	amine metabolic process	0.0004
Clayton	F_1_>N7103	GO:0043531	ADP binding	0.000081
Clayton	F_1_<N7103	GO:0071555	cell wall organization	0.00017
Clayton	F_1_<N7103	GO:0055114	oxidation reduction	0.00041
Clayton	F_1_<N7103	GO:0030599	pectinesterase activity	6.7E-10
Clayton	F_1_<N7103	GO:0003682	chromatin binding	0.00088
Clayton	F_1_>NMS4-44-329	GO:0008202	steroid metabolic process	0.000052
Clayton	F_1_>NMS4-44-329	GO:0006694	steroid biosynthetic process	0.00045
Clayton	F_1_<NMS4-44-329	GO:0055114	oxidation reduction	0.0000086
Clayton	F_1_<NMS4-44-329	GO:0030599	pectinesterase activity	0.00003
Clayton	F_1_<NMS4-44-329	GO:0004857	enzyme inhibitor activity	0.00012
Clayton	F_1_<> both	GO:0030599	pectinesterase activity	0.00000029
Clayton	F_1_<> both	GO:0004857	enzyme inhibitor activity	0.0000013
Clayton	F_1_<> both	GO:0016491	oxidoreductase activity	0.0015

Selected gene ontologies that were enriched in recently expanded trifoliates harvested from Kinston or Clayton. Expression refers to the same categories as Table 4 except “<>both” is all genes with non-additive expression.

Expression of most genes in the F_1_ hybrids fell between the parental values. We interpreted this to mean that differential expression of these genes was largely additive. Recombining additive gene expression patterns is likely to be the basis of a substantial number of inherited phenotypes. However, analyses of genes up-regulated or down-regulated between the F_1_ hybrids and both parents identified a unique category of expression that was not additive. These genes were expressed at a lower or higher level in the F_1_ hybrid than both parents and the number of genes falling into these categories is shown in Table 4. This category accounts for less than 0.1% of genes expressed in leaves. At Kinston, 25 genes were expressed at a higher level in the F_1_ hybrid than in either parent and 6 genes were expressed a lower level (Table 6). The Gene ID, the fold change in expression and information about the possible function of these genes, are shown in Table 6. Of note is how few of these genes were well annotated. Two genes, Glyma.03G016300 and Glyma.03G016400, are 68% identical and are associated with ethylene biosynthesis. Both genes were expressed at a higher level in the F_1_ hybrid than in either parent. Three calcium-binding proteins were up-regulated in the F_1_ hybrids while one calcium-binding gene was down regulated. Two of the up-regulated calcium-binding genes, Glyma.20g034200 and Glyma.07g229500, are over 75% identical to each other. Glyma.20g034200 and Glyma.07g229500 are annotated as a class of calcium binding proteins important in signal transduction called calmodulins.

**Table 6:** Non-additive gene expression in Kinston.

ID score	Sequence ID	EXP	Function
0	Glyma.08G085700	-51.41	Uncharacterized
0	Glyma.01G078700	-5.6	Uncharacterized
2(68%)	Glyma.03G016300	-3.06	ethylene biosynthetic
1(75%)	Glyma.20G034200	-2.77	Calmodulin
1(75%)	Glyma.07G229500	-2.76	Calmodulin
2(68%)	Glyma.03G016400	-2.21	ethylene biosynthetic
0	Glyma.11G124700	-2.19	Uncharacterized
0	Glyma.05G077300	-2.07	Uncharacterized
0	Glyma.15G270000	-1.98	Uncharacterized
0	Glyma.15G270100	-1.86	Siroheme Synthase
0	Glyma.05G211600	-1.85	Uncharacterized
0	Glyma.13G042200	-1.74	flavanone 3-hydroxylase
0	Glyma.15G198900	-1.58	Uncharacterized
0	Glyma.03G197800	-1.49	Uncharacterized
0	Glyma.06G057800	-1.42	Uncharacterized
0	Glyma.14G069400	-1.41	Uncharacterized
0	Glyma.06G258000	-1.39	calcium binding
0	Glyma.09G016400	-1.34	metal transport
0	Glyma.17G058300	-1.32	Monoacylglycerol acyltransferase
0	Glyma.02G168600	-1.29	Uncharacterized
0	Glyma.15G098700	-1.25	Uncharacterized
0	Glyma.02G125800	-1.2	Uncharacterized
0	Glyma.20G147800	-1.1	Estradiol 17-beta-dehydrogenase
0	Glyma.06G301100	-1.1	Methyltransferase
0	Glyma.04G059400	-1.07	Uncharacterized
0	Glyma.13G353500	1.1	Mitochondrial-processing peptidase
0	Glyma.07G010000	1.11	Uncharacterized
0	Glyma.14G014600	1.35	Pectinesterase
0	Glyma.17G039400	1.39	Uncharacterized
0	Glyma.19G151600	1.87	Uncharacterized
0	Glyma.11G056200	2.44	Heat shock transcription factor

Sequence comparisons of genes with a “non-additive” pattern of expression in Kinston. Identity refers to genes that share amino sequence identity, a 0 means no sequences with > 20% identity were identified, the value in parenthesizes is the identity shared by similar non-additively expressed sequences. The longest protein sequence for each gene model present in soybase was used for the alignment. Sequence ID is the gene model from soybase. Expression (EXP) is the average fold change between Kinston and Clayton. Function was selected from the soybase, and was the most likely informative option available in soybase.

At Clayton, 22 genes were expressed at higher levels and 35 genes were expressed at lower levels in the F_1_ hybrid relative to either parent, Table 4. Table 7 shows information about the non-additively expressed genes in Clayton. Many genes down-regulated in the F_1_ hybrid were annotated as cell wall related and pectin methyl esterase inhibitors that may be associated with cell expansion. Four genes annotated as asparagine synthases are up-regulated in the hybrid. Alignment of these asparagine synthases sequences revealed that three of them, Glyma.15G071300, Glyma.12G150500 and Glyma.13G181000, are between 62 and 82% identical. However, the other asparagine synthase gene, Glyma.11G171400, is less than 20% identical to the other three. Transcripts of 3 myo-inositol oxygenases, encoding proteins ranging from 68% to 72% identical, increased in expression in the F_1_ hybrids. Other related genes also appeared to be differentially expressed in the hybrid, but as a family their pattern was hard to discern. For example, three genes with similar annotations, Glyma.15G093900, Glyma.11g230800, Glyma.11g027700 were differentially regulated in the F_1_ hybrids. Glyma.15G093900 may be a Gibberellin 2-beta-dioxygenase and was increased in expression in the F_1_ hybrids. The other 2 genes decreased in expression in the F_1_ hybrids and may be Leucoanthocyanidin dioxygenase. These three digoxygenases range from 24 to 34% identical. Alignment of proteins encoded by genes with nonadditive patterns of expression has identified poorly annotated genes that share substantial identity with other genes on the list. Glyma.15G211300 and Glyma.03G113200 are 96% identical. Glyma.08G081800 and Glyma.05G126800 are 78% identical. Glyma.10G248900 and Glyma.20G144800 share 86% identity. Glyma.10G130600 and Glyma.20G081400 share 93% identity and are annotated as LEA protein. Glyma.10G168500, Glyma.03G252600 and Glyma.19G250100 are 73-97% identical and are annotated as GDSL like lipases.

**Table 7:** Non-additive gene expression in Clayton.

ID score	Sequence ID	EXP	Function
0	Glyma.09G080100	-4.43	Cytosine_deaminase
7(62-82%)	Glyma.15G071300	-3.89	Asparagine synthetase
2(96%)	Glyma.15G211300	-3.84	Uncharacterized
0	Glyma.19G239800	-2.84	Shikimate dehydrogenase
0	Glyma.11G171400	-2.53	Asparagine synthetase
1(69-72%)	Glyma.01G005100	-2.5	myo-inositol oxygenase
0	Glyma.15G062400	-2.33	Uncharacterized
0	Glyma.17G092800	-2.03	regulation of reactive oxygen
1(69-72%)	Glyma.05G224500	-1.98	myo-inositol oxygenase
2(96%)	Glyma.03G113200	-1.94	Uncharacterized
7(62-82%)	Glyma.12G150500	-1.94	Asparagine synthetase
4(86%)	Glyma.10G248900	-1.82	haloacid_dehalogenase-like_hydrolase
7(62-82%)	Glyma.13G181000	-1.76	Asparagine synthetase
0	Glyma.14G010500	-1.64	Raffinose Synthase
0	Glyma.06G050100	-1.54	amino-acid transaminase
1(69-72%)	Glyma.08G199300	-1.41	myo-inositol oxygenase
4(86%)	Glyma.20G144800	-1.37	haloacid_dehalogenase-like_hydrolase
0	Glyma.15G023800	-1.15	carbohydrate binding
0	Glyma.04G018400	-1.07	Uncharacterized
9(27-23%)	Glyma.15G093900	-1.06	gibberellin 2-beta-dioxygenas
6(97-73%)	Glyma.10G168500	1	GDSL-like Lipase/Acylhydrolase
0	Glyma.02G008300	1.04	pectinesterase activity
0	Glyma.03G262600	1.05	haloacid dehalogenase-like hydrolase
0	Glyma.10G246600	1.06	Uncharacterized
0	Glyma.18G199300	1.12	starch binding
5(93%)	Glyma.10G130600	1.14	LEA
0	Glyma.07G225400	1.2	Multicopper_oxidases
5(93%)	Glyma.20G081400	1.22	LEA
0	Glyma.09G021900	1.26	Invertase/PME inhibitor
0	Glyma.18G214600	1.27	lipid binding
0	Glyma.13G299400	1.28	Uncharacterized
0	Glyma.04G204900	1.33	Uncharacterized
9(35%)	Glyma.11G027700	1.34	Leucoanthocyanidin dioxygenase
0	Glyma.08G030500	1.35	caffeoyl-CoA O-methyltransferase
0	Glyma.10G223100	1.35	Uncharacterized
0	Glyma.20G208300	1.38	lipid binding
0	Glyma.20G146700	1.44	Uncharacterized
6(97-73%)	Glyma.03G252600	1.45	GDSL-like Lipase/Acylhydrolase
0	Glyma.07G011700	1.45	haloacid_dehalogenase-like_hydrolase
0	Glyma.18G240000	1.52	Uncharacterized
0	Glyma.13G328400	1.53	nutrient reservoir activity
0	Glyma.08G211600	1.54	AP2 domain
0	Glyma.19G025000	1.58	MYB domain
0	Glyma.19G186700	1.63	cell wall loosening
0	Glyma.19G212200	1.65	Uncharacterized
6(97-73%)	Glyma.19G250100	1.67	GDSL-like Lipase/Acylhydrolase
0	Glyma.02G180200	1.69	Uncharacterized
8 (38%)	Glyma.15G082300	1.84	cell wall modification
0	Glyma.05G126800	2.01	NOD 19
8(38%)	Glyma.13G270700	2.2	cell wall modification
0	Glyma.04G199600	2.7	GDSL-like Lipase/Acylhydrolase

Gene ontologies and sequence comparisons of genes with a “non-additive” pattern of expression in Clayton. Identity refers to genes that share amino sequence identity, a 0 means no sequences > 20% identical were identified, the value in parenthesizes is the identity shared by similar non-additively expressed sequences. The longest protein sequence for each gene model present in soybase was used for the alignment. Sequence ID is the gene model from soybase. Expression is the average fold change between Kinston and Clayton, negative values indicate higher expression in the hybrid. Function was selected from soybase, and was the most likely informative option available in soybase.

### Allele specific bias

**Fig 1:**
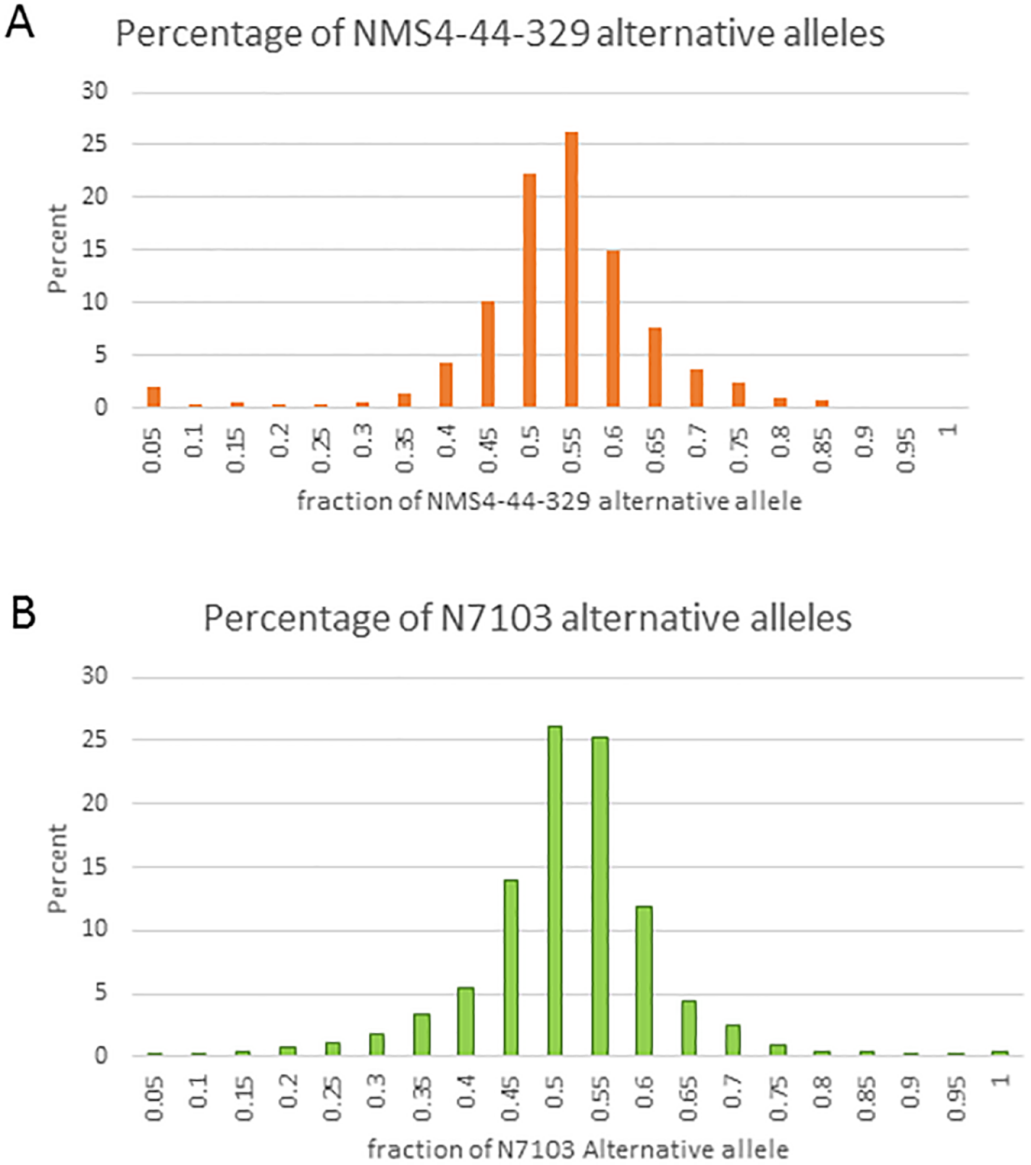
The distribution of the bias of alternative SNPs. The fraction of the alternative allele is shown as the percent of total SNPS. Panel A shows the alternative allele (relative to the reference genome) from NMS4-44-329 and Panel B Shows the alternative alleles derived from N7103. Low values (>0.5) indicate higher levels of expression of the SNP from NMS4-44-329.

One advantage of using NMS4-44-329 as a parent is that alleles/genetic regions inherited from *G*. *soja* are likely to have more polymorphic SNPs than alleles inherited from a typical *G*. *max* genotype. It is possible that some SNPs occur *de novo* in the recurrent parent, N7103, but it is likely that most SNPs were inherited from the wild grandparent (PI 366122). In most cases, total gene expression is represented by about a 50% contribution of each allele, although in some cases allele specific expression can occur. We defined allele specific bias as one allele representing over 73% of total expression which is greater than 2 standard deviations in this experiment. Over 4390 high quality SNPs representing about 3,157 genes were identified that contrast between N7103 and NMS4-44-329 in the RNAseq data. The total read counts of each SNP were measured in the F_1_ hybrids and the ratio of the counts from each SNP determined. Unlike differential gene expression in the F_1_ hybrids, the ratio of alleles expressed in the F_1_ hybrid for each high quality SNP was well correlated between Kinston and Clayton (r^2^ = 0.49). The correlation of gene expression bias greater than or equal to two standard deviations between locations was extremely well correlated (r^2^ = 0.97). No allele switched bias between locations. Only 241 SNPs, shown in S2 Table, had expression biases of greater than or equal to 2 standard deviations. These 241SNPs represented variants in 191 genes. Of the 29 genes represented by more than one SNP, only three (noted by an asterisk in S2 Table) were inconsistent in their allele biases. We note that the NMS4-44-329 alternative alleles were down regulated more than the N7103 alleles. One explanation for this bias was that the NMS4-44-329 plants used in the hybridization were derived from a single plant in the F_4_ generation. Due to residual heterozygosity (expected 12.5%) in the F_4_ plant selection, a heterogeneous seed-lot would have been produced through further self-pollination, so a small amount of genetic variation would have existed between NMS4-44-329 plants used for hybridization. As a result, some F_1_ hybrids could be lacking an allele based on the NMS4-44-329 parent lacking the allele. To address this concern, alleles were only used that were not polymorphic in any parental line sequenced. The distribution of alleles is shown in Fig 1. The bins with lower ratios of the NMS4-44-329 alleles were shifted to slightly higher percentages. This might reflect a shift toward reduced expression of NMS4-44-329 alleles in a predominantly N7103 genetic background or a slight underestimation of NMS4-44-329 alleles due to some residual heterozygosity in the original parent as described above. In rice an increased frequency of C to T and G to A SNPs has been reported that is a consequences of the higher frequency of mutation of methylcytosine [24]. We investigated the frequency of this class of SNPs among alleles with a biased gene expression because methylcytosines are epigenetic marks capable of affecting transcription. However, there was no significant difference in the frequency of relevant SNPs between genes with biased expression and those without. The SEA analyses of the ontology of genes displaying expression bias indicated that genes associated with “adenyl ribonucleotide binding” were significantly enriched (3.4X10^-6^) among genes biased in both directions. No genes were identified with non-additive expression and an allele bias.

## Discussion

One goal of this research was to determine whether or not the presence of wild soybean in the pedigree of a parent would lead to heterosis in the progeny. Reported estimates of heterosis for seed yield, and other agronomic traits are generally small in soybean and many other self-pollinated crops (often less than 20%), as compared to cross pollinated species such as corn [9]. However, the F_1_-hybrid had numerically improved yields, seed protein content, and seed protein production per hectare relative to the mid-parent and each parent at each location. Seed weight was also greater for the F_1_ hybrid than for the mid-parent value at each location. All of these measures of increased productivity for the F_1_ hybrid were consistent with heterosis. Inbreeding depression, another hallmark of heterosis was noted for yield in the F_2_ populations at each location, as compared to the F_1_. However, no inbreeding depression was noted for seed protein content suggesting that the F_1_ heterosis may be derived from epistasis rather than dominance for that trait. To our knowledge this is the first report of heterosis for seed protein content of soybean.

Comparison of seed yield, seed protein content, and seed protein production per hectare in the hybrids and parents suggested that alleles from the wild soybean are potential sources for improvement of agronomic traits. The yield of the F_1_ was similar to that of modern elite cultivar N7003CN of similar maturity, indicating that the yield levels in this study were sufficiently high to suggest that the *G*. *soja* derived alleles may have important agronomic potential in plant breeding. The frequently reported negative correlation between seed protein content and seed yield was not observed in this study, providing a rare opportunity to improve both yield and seed protein content simultaneously, using alleles from the wild soybean [25].

The second goal of this research was to compare genes differentially expressed in the leaves of the F_1_ hybrid to that of the parents to gain insight into the physiological basis of the yield increase. Identifying genes associated with improved yield was challenging because this is a complex trait, there was a strong environmental effect and the increase in yield of the F_1_ hybrids compared to the mid-parent was small. Comparison of the ontologies enriched in each location relative to the mid-parent expression (F_1_>both or F_1_<both in Table 4) failed to identify a GO term consistently enriched or depleted in the F_1_ compared to the parents. The biggest improvement in yield compared to the hybrid was between NMS4-44-329 in Kinston (270 kg ha-1) and N7103 in Clayton (307 Kg ha-1). No clear overlap is observed in the ontologies enriched in the F_1_ hybrid compared to NMS4-44-329 in Kinston and N7103 in Clayton. Genes with a non-additive pattern of expression could play an important role in yield improvement of the F_1_ hybrids. In many cases families of genes that share substantial identity and presumably similar functions shared a non-additive pattern of expression. Analyses of these genes identified good candidates to impact a complicated trait like yield. For example, calmodulin plays a role in transmitting signal cascades and remodeling calmodulin expression could impact multiple traits [26,27]. One gene had a non-additive pattern of expression in the F_1_ hybrids compared to NMS4-44-329 in Kinston and N7103 in Clayton. This gene is a methionine sulfoxide reductase annotated as being involved more broadly in oxidation reduction. This gene has been well studied in *G*. *soja* as important in tolerance to alkaline stress [28]. Perhaps it plays a role in improving yield as well. Genes with an allele specific expression bias in the F_1_ hybrids were enriched for “ADP-binding proteins”. We note that this category overlaps a category enriched in high yielding F_1_ hybrids in Clayton. Therefore, the impact of “ADP-binding proteins” on yield may be worth further investigation.

The third goal of this research was to compare genes differentially expressed in the leaves of the F_1_ hybrid relative to the parents to gain insight into the physiological basis of improved seed protein content. The improvement of the protein content of seed and seed production per hectare derived from the F_1_ hybrids over the mid-parent value in both locations has provided a robust dataset to identify genes associated with improved protein content in soybean. Genes associated with the “amine metabolic process” that could potentially impact N metabolism in the leaf and may ultimately impact seed protein content were up-regulated in leaves in one location. Four asparagine synthetase genes were increased in expression in the F_1_ compared to both parents in Clayton. Asparagine and asparagine synthase are thought to play an important role in N use efficiency and alterations in this metabolic pathway in the leaves could impact seed composition by affecting N availability [29–31]. In addition to four asparagine synthase genes, three of which were highly identical, 15 other genes were non-additively expressed with closely related genes based on sequence identity. Co-expression of orthologous sequences is consistent with the proposal of Burton and Brownie that duplicate gene interactions may be associated with heterosis in soybean. Evaluation of the progeny of the F_1_ hybrids for elevated asparagine synthetase expression may provide a method to identify the best lines for elevated seed protein early in the breeding process. Some differentially expressed genes in the relevant categories discussed above are not represented in enriched ontologies. For example, one of the genes with an allele specific bias is annotated as a nitrate transporter that may play a role in improving N balance. The inheritance and expression of these genes can be evaluated in the progeny of the F_1_ hybrids.

This research suggests that additive gene expression, non-additive gene expression and allele specific expression bias could be used to evaluate the likelihood that F_1_ hybrids will produce agronomically valuable progeny. Non-additive gene expression reported for maize heterotic hybrids relative to their parents appeared to be more common than we report for soybean [32,33]. Historically, genetic markers linked to difficult to select traits and markers associated with QTLs have played an important role in plant breeding. Sequencing based approaches are no longer uncommon in plant breeding, and with less expensive sequencing, it is worth considering using sequencing to evaluate crosses early in the breeding process. F_1_ hybrids are generated in the breeding process and are available for nondestructive analyses. Creating these initial crosses is not trivial, but it is not the most difficult or expensive part of the breeding process. If multiple promising parents are selected it may be possible to pick the most promising crosses for further analyses by evaluating the parameters listed above. Future evaluation of progeny between NMS4-44-329 and N7103 will provide a test case to determine if increased expression of asparagine synthase is associated with improve seed protein content in multiple environments and if this pattern of expression persists during seed fill when seed protein content is likely to be impacted. One limitation of this approach is the location specific expression pattern of many of the relevant genes. If the expression of genes varies across years or locations, they may be difficult to associate with the desired trait. Therefore it is worth focusing on changes in gene expression that are not affected by the environment. As the correlation between gene expression and phenotypes are investigated in more environments, it may be possible to gain insight into physiological pathways associated with traits influenced by environmental effects by evaluating changes in gene expression that are not similar across environments. In this study, the allele specific bias was also very stable across locations. In cases where this bias is associated with a targeted trait it may be possible to use this biased expression as leverage for progeny selection in a breeding program.

## Supporting information

S1 Table Raw data used to calculate agronomic information. (XLSX)

S2 Table Allele specific bias of soybean gene models. (DOCX)
