# Supplementary material for: Changes in gene expression between a soybean F1 hybrid and its parents are associated with agronomically valuable traits
Source: PLoS One. 2017 May 11;12(5):e0177225. doi: 10.1371/journal.pone.0177225 (PMC5426663; doi:10.1371/journal.pone.0177225)
Supplement: S2 Table — (DOCX) [file pone.0177225.s002.docx]

Supplementary data 2.

Allele Specific Bias

| Gene ID | REF | ALT | reference allele | Clayton | Kinston |
| --- | --- | --- | --- | --- | --- |
| Glyma.01G009300 | T | G | NMS4-44-329 | 0.00 | 0.00 |
| Glyma.01G089600 | A | T | N7103 | 0.27 | 0.23 |
| Glyma.01G089900 | C | A | N7103 | 0.17 | 0.18 |
| Glyma.02G048300 | G | A | N7103 | 0.05 | 0.00 |
| Glyma.02G226900 | G | A | NMS4-44-329 | 0.87 | 0.80 |
| Glyma.02G235100 | A | G | NMS4-44-329 | 0.78 | 0.72 |
| Glyma.02G277300 | A | T | NMS4-44-329 | 0.78 | 0.81 |
| Glyma.02G291700 | A | T | NMS4-44-329 | 0.25 | 0.17 |
| Glyma.03G021100 | C | A | N7103 | 0.84 | 0.77 |
| Glyma.03G032700 | G | A | N7103 | 0.13 | 0.02 |
| Glyma.03G033300 | T | A | N7103 | 0.00 | 0.24 |
| Glyma.03G034400 | G | T | NMS4-44-329 | 0.00 | 0.00 |
| Glyma.03G043400 | C | T | N7103 | 0.77 | 0.71 |
| Glyma.03G043700* | G | A | N7103 | 0.18 | 0.02 |
| Glyma.03G043700* | G | A | N7103 | 0.86 | 0.83 |
| Glyma.03G047000 | A | T | NMS4-44-329 | 0.21 | 0.00 |
| Glyma.03G047100 | C | G | N7103 | 0.00 | 0.01 |
| Glyma.03G101200 | G | A | N7103 | 0.77 | 0.81 |
| Glyma.03G137000 | A | G | NMS4-44-329 | 0.73 | 0.76 |
| Glyma.03G148300 | C | A | N7103 | 0.20 | 0.01 |
| Glyma.03G151200 | A | G | N7103 | 0.04 | 0.04 |
| Glyma.03G179800 | T | A | NMS4-44-329 | 0.71 | 0.76 |
| Glyma.03G211000 | G | A | NMS4-44-329 | 0.96 | 0.98 |
| Glyma.03G250800 | C | T | N7103 | 0.77 | 0.78 |
| Glyma.04G092300 | A | G | NMS4-44-329 | 0.75 | 0.86 |
| Glyma.04G123900 | A | G | N7103 | 0.84 | 0.80 |
| Glyma.04G194500 | C | T | NMS4-44-329 | 0.70 | 0.76 |
| Glyma.05G003900 | T | A | N7103 | 0.05 | 0.01 |
| Glyma.05G004100 | C | T | N7103 | 0.75 | 0.81 |
| Glyma.05G006700 | A | G | N7103 | 0.80 | 0.84 |
| Glyma.05G010400 | C | T | N7103 | 0.82 | 0.81 |
| Glyma.05G023000 | A | G | NMS4-44-329 | 0.29 | 0.22 |
| Glyma.05G030000 | T | C | N7103 | 0.00 | 0.00 |
| Glyma.05G031100 | T | A | NMS4-44-329 | 0.02 | 0.00 |
| Glyma.05G031100 | G | T | NMS4-44-329 | 0.00 | 0.00 |
| Glyma.05G059300 | T | C | N7103 | 0.78 | 0.86 |
| Glyma.05G082200 | T | C | NMS4-44-329 | 0.80 | 0.70 |
| Glyma.05G087100 | A | T | N7103 | 0.00 | 0.00 |
| Glyma.05G105000 | T | G | NMS4-44-329 | 0.76 | 0.85 |
| Glyma.05G117900 | A | G | N7103 | 0.22 | 0.18 |
| Glyma.05G131200 | A | G | NMS4-44-329 | 0.77 | 0.84 |
| Glyma.05G133100 | T | A | NMS4-44-329 | 0.73 | 0.84 |
| Glyma.05G236600 | T | C | N7103 | 0.00 | 0.00 |
| Glyma.06G200000 | T | C | NMS4-44-329 | 0.26 | 0.23 |
| Glyma.07G014300 | G | C | NMS4-44-329 | 0.13 | 0.19 |
| Glyma.07G026800 | A | G | NMS4-44-329 | 0.71 | 0.83 |
| Glyma.07G062900* | G | C | NMS4-44-329 | 0.78 | 0.75 |
| Glyma.07G062900* | A | G | NMS4-44-329 | 0.20 | 0.17 |
| Glyma.07G062900* | T | A | NMS4-44-329 | 0.26 | 0.13 |
| Glyma.07G062900* | A | C | NMS4-44-329 | 0.83 | 0.85 |
| Glyma.07G063300 | C | T | NMS4-44-329 | 0.74 | 0.76 |
| Glyma.07G063600 | G | T | NMS4-44-329 | 0.24 | 0.25 |
| Glyma.07G064100 | T | C | NMS4-44-329 | 0.77 | 0.82 |
| Glyma.07G064100 | G | C | NMS4-44-329 | 0.85 | 0.87 |
| Glyma.07G064100 | A | G | NMS4-44-329 | 0.76 | 0.79 |
| Glyma.07G064100 | G | A | NMS4-44-329 | 0.84 | 0.74 |
| Glyma.07G064100 | G | A | NMS4-44-329 | 0.75 | 0.71 |
| Glyma.07G064100 | G | C | NMS4-44-329 | 0.76 | 0.80 |
| Glyma.07G064900 | G | C | NMS4-44-329 | 0.84 | 0.85 |
| Glyma.07G064900 | G | C | NMS4-44-329 | 0.96 | 0.94 |
| Glyma.07G064900 | C | T | NMS4-44-329 | 0.80 | 0.75 |
| Glyma.07G064900 | C | A | NMS4-44-329 | 0.90 | 0.91 |
| Glyma.07G064900 | C | T | NMS4-44-329 | 0.92 | 0.93 |
| Glyma.07G065000* | C | T | NMS4-44-329 | 0.00 | 0.02 |
| Glyma.07G065000* | G | A | NMS4-44-329 | 0.04 | 0.02 |
| Glyma.07G065000* | C | G | N7103 | 0.05 | 0.01 |
| Glyma.07G065000* | A | G | N7103 | 0.06 | 0.15 |
| Glyma.07G065000* | G | T | NMS4-44-329 | 0.76 | 0.76 |
| Glyma.07G065500 | T | A | N7103 | 0.19 | 0.14 |
| Glyma.07G065500 | G | C | NMS4-44-329 | 0.00 | 0.01 |
| Glyma.07G065600 | A | G | NMS4-44-329 | 0.80 | 0.76 |
| Glyma.07G065600 | G | A | NMS4-44-329 | 0.75 | 0.80 |
| Glyma.07G090200 | A | G | NMS4-44-329 | 0.89 | 0.88 |
| Glyma.07G091500 | A | G | NMS4-44-329 | 0.80 | 0.76 |
| Glyma.07G096600 | A | G | N7103 | 0.00 | 0.00 |
| Glyma.07G130200 | G | A | N7103 | 0.86 | 0.91 |
| Glyma.07G156900 | T | C | N7103 | 0.00 | 0.00 |
| Glyma.07G157000 | G | C | N7103 | 0.00 | 0.00 |
| Glyma.07G157200 | T | G | N7103 | 0.00 | 0.00 |
| Glyma.07G193700 | T | C | N7103 | 0.00 | 0.00 |
| Glyma.07G201300 | G | T | NMS4-44-329 | 0.98 | 0.97 |
| Glyma.07G201900 | A | T | NMS4-44-329 | 0.06 | 0.20 |
| Glyma.07G204700 | T | G | N7103 | 0.83 | 0.83 |
| Glyma.07G204700 | T | A | N7103 | 0.83 | 0.81 |
| Glyma.07G247900 | G | A | N7103 | 0.76 | 0.72 |
| Glyma.08G040000 | G | C | NMS4-44-329 | 0.84 | 0.87 |
| Glyma.08G040800 | G | C | NMS4-44-329 | 0.85 | 0.90 |
| Glyma.08G085900 | A | G | N7103 | 0.79 | 0.80 |
| Glyma.08G114200 | G | C | N7103 | 0.93 | 0.94 |
| Glyma.08G123500 | T | G | N7103 | 0.17 | 0.25 |
| Glyma.08G123800 | T | A | N7103 | 0.11 | 0.00 |
| Glyma.08G124900 | A | T | NMS4-44-329 | 0.82 | 0.82 |
| Glyma.08G135000 | A | G | NMS4-44-329 | 0.73 | 0.78 |
| Glyma.08G145200 | A | C | N7103 | 0.04 | 0.00 |
| Glyma.08G146200 | A | G | NMS4-44-329 | 0.71 | 0.82 |
| Glyma.08G317700 | C | A | NMS4-44-329 | 0.23 | 0.24 |
| Glyma.08G318400 | G | A | NMS4-44-329 | 0.19 | 0.15 |
| Glyma.08G320700 | T | A | N7103 | 0.00 | 0.00 |
| Glyma.08G321000 | T | C | N7103 | 0.00 | 0.03 |
| Glyma.08G325000 | T | C | NMS4-44-329 | 0.00 | 0.00 |
| Glyma.08G336300 | G | A | N7103 | 0.00 | 0.00 |
| Glyma.08G339400 | G | A | NMS4-44-329 | 0.90 | 0.83 |
| Glyma.08G342300 | A | C | N7103 | 0.15 | 0.28 |
| Glyma.08G343800 | A | C | NMS4-44-329 | 0.92 | 0.97 |
| Glyma.08G347800 | C | G | NMS4-44-329 | 0.77 | 0.70 |
| Glyma.08G351800 | C | T | NMS4-44-329 | 0.14 | 0.05 |
| Glyma.08G351800 | T | C | NMS4-44-329 | 0.07 | 0.04 |
| Glyma.08G351800 | C | T | NMS4-44-329 | 0.19 | 0.00 |
| Glyma.08G355000 | A | T | NMS4-44-329 | 0.19 | 0.25 |
| Glyma.09G005700 | G | T | N7103 | 0.84 | 0.82 |
| Glyma.09G051100 | A | G | NMS4-44-329 | 0.81 | 0.77 |
| Glyma.09G057400 | T | A | NMS4-44-329 | 0.09 | 0.00 |
| Glyma.09G065000 | C | A | N7103 | 0.77 | 0.74 |
| Glyma.09G071300 | G | C | NMS4-44-329 | 0.77 | 0.82 |
| Glyma.09G091200 | T | G | N7103 | 0.87 | 0.85 |
| Glyma.09G162100 | C | G | NMS4-44-329 | 0.75 | 0.77 |
| Glyma.09G196000 | T | A | NMS4-44-329 | 0.95 | 0.98 |
| Glyma.09G196200 | T | C | NMS4-44-329 | 0.88 | 0.89 |
| Glyma.09G196200 | C | T | NMS4-44-329 | 0.81 | 0.82 |
| Glyma.09G208900 | G | T | NMS4-44-329 | 0.07 | 0.22 |
| Glyma.09G208900 | T | C | NMS4-44-329 | 0.24 | 0.13 |
| Glyma.09G209100 | C | G | NMS4-44-329 | 0.83 | 0.80 |
| Glyma.09G209100 | G | A | NMS4-44-329 | 0.85 | 0.84 |
| Glyma.09G209100 | A | C | NMS4-44-329 | 0.84 | 0.86 |
| Glyma.09G210700 | G | A | NMS4-44-329 | 0.72 | 0.75 |
| Glyma.09G270500 | G | A | NMS4-44-329 | 0.76 | 0.74 |
| Glyma.10G013300 | T | G | NMS4-44-329 | 0.91 | 0.92 |
| Glyma.10G014200 | C | T | NMS4-44-329 | 0.79 | 0.80 |
| Glyma.10G211600 | A | T | N7103 | 0.22 | 0.23 |
| Glyma.10G215400 | C | T | NMS4-44-329 | 0.74 | 0.81 |
| Glyma.10G215400 | T | C | NMS4-44-329 | 0.72 | 0.80 |
| Glyma.10G215400 | G | A | NMS4-44-329 | 0.71 | 0.80 |
| Glyma.11G067800 | G | A | NMS4-44-329 | 0.83 | 0.82 |
| Glyma.11G068000 | C | T | NMS4-44-329 | 0.77 | 0.81 |
| Glyma.11G102600 | T | C | NMS4-44-329 | 0.82 | 0.79 |
| Glyma.11G107500 | G | A | NMS4-44-329 | 0.85 | 0.78 |
| Glyma.11G136100 | T | A | NMS4-44-329 | 0.82 | 0.79 |
| Glyma.11G145600 | A | C | NMS4-44-329 | 0.76 | 0.80 |
| Glyma.11G151100 | A | G | NMS4-44-329 | 0.75 | 0.73 |
| Glyma.11G155300 | T | C | N7103 | 0.00 | 0.00 |
| Glyma.11G155300 | G | A | N7103 | 0.00 | 0.00 |
| Glyma.11G160000 | G | A | N7103 | 0.97 | 0.97 |
| Glyma.11G170200 | T | C | N7103 | 0.00 | 0.00 |
| Glyma.11G189000 | G | A | NMS4-44-329 | 0.80 | 0.86 |
| Glyma.11G190700 | A | C | N7103 | 0.78 | 0.78 |
| Glyma.11G214800 | A | C | N7103 | 0.75 | 0.72 |
| Glyma.11G214800 | G | T | N7103 | 0.77 | 0.77 |
| Glyma.12G195100 | T | C | N7103 | 0.00 | 0.00 |
| Glyma.13G000600 | C | T | N7103 | 0.21 | 0.13 |
| Glyma.13G000900 | C | T | N7103 | 0.14 | 0.07 |
| Glyma.13G001000 | G | C | N7103 | 0.06 | 0.10 |
| Glyma.13G032500 | G | A | N7103 | 0.00 | 0.00 |
| Glyma.13G032700 | T | G | N7103 | 0.00 | 0.00 |
| Glyma.13G032900 | A | G | N7103 | 0.07 | 0.06 |
| Glyma.13G034000 | G | A | N7103 | 0.00 | 0.00 |
| Glyma.13G034000 | T | C | N7103 | 0.00 | 0.00 |
| Glyma.13G034500 | G | T | N7103 | 0.00 | 0.00 |
| Glyma.13G035900 | A | G | N7103 | 0.04 | 0.01 |
| Glyma.13G035900 | G | A | NMS4-44-329 | 0.00 | 0.00 |
| Glyma.13G175300 | T | C | NMS4-44-329 | 0.82 | 0.87 |
| Glyma.13G175300 | T | G | NMS4-44-329 | 0.93 | 0.95 |
| Glyma.13G177800 | C | G | NMS4-44-329 | 0.88 | 0.88 |
| Glyma.13G177800 | G | A | NMS4-44-329 | 0.87 | 0.89 |
| Glyma.13G195100 | G | A | N7103 | 0.18 | 0.27 |
| Glyma.13G198000 | G | A | NMS4-44-329 | 0.00 | 0.00 |
| Glyma.13G245900 | G | T | NMS4-44-329 | 0.79 | 0.75 |
| Glyma.13G245900 | C | G | NMS4-44-329 | 0.87 | 0.83 |
| Glyma.13G280300 | G | A | NMS4-44-329 | 0.80 | 0.84 |
| Glyma.13G297800 | C | T | NMS4-44-329 | 0.23 | 0.26 |
| Glyma.13G297900 | A | T | NMS4-44-329 | 0.15 | 0.11 |
| Glyma.13G298300 | G | A | N7103 | 0.00 | 0.00 |
| Glyma.13G299300 | A | G | N7103 | 0.79 | 0.72 |
| Glyma.13G300000 | A | T | N7103 | 0.74 | 0.73 |
| Glyma.13G341500 | C | G | N7103 | 0.74 | 0.76 |
| Glyma.14G205000 | A | G | N7103 | 0.00 | 0.00 |
| Glyma.14G205100 | G | A | N7103 | 0.00 | 0.00 |
| Glyma.14G206500 | T | G | N7103 | 0.00 | 0.01 |
| Glyma.14G206500 | T | A | N7103 | 0.00 | 0.03 |
| Glyma.14G207700 | T | C | N7103 | 0.06 | 0.03 |
| Glyma.14G209800 | C | T | N7103 | 0.84 | 0.91 |
| Glyma.15G012500 | T | C | N7103 | 0.78 | 0.77 |
| Glyma.15G021400 | C | T | N7103 | 0.21 | 0.09 |
| Glyma.15G021400 | A | G | N7103 | 0.16 | 0.07 |
| Glyma.15G021400 | A | G | N7103 | 0.18 | 0.12 |
| Glyma.15G021700 | T | C | N7103 | 0.08 | 0.08 |
| Glyma.15G021700 | T | A | N7103 | 0.20 | 0.14 |
| Glyma.15G022100 | C | A | N7103 | 0.07 | 0.04 |
| Glyma.15G057700 | C | G | N7103 | 0.77 | 0.80 |
| Glyma.16G025800 | T | C | N7103 | 0.72 | 0.81 |
| Glyma.16G073300 | T | G | NMS4-44-329 | 0.77 | 0.71 |
| Glyma.16G167500 | T | C | NMS4-44-329 | 0.22 | 0.28 |
| Glyma.16G172500 | T | G | N7103 | 0.16 | 0.25 |
| Glyma.16G179100 | C | T | N7103 | 0.85 | 0.83 |
| Glyma.16G196800 | T | C | NMS4-44-329 | 0.00 | 0.03 |
| Glyma.16G197100 | T | C | N7103 | 0.77 | 0.72 |
| Glyma.16G198500 | T | G | NMS4-44-329 | 0.73 | 0.77 |
| Glyma.16G202000 | A | T | NMS4-44-329 | 0.00 | 0.00 |
| Glyma.16G208100 | T | C | NMS4-44-329 | 0.76 | 0.74 |
| Glyma.16G209100 | T | A | NMS4-44-329 | 0.27 | 0.21 |
| Glyma.16G213700 | G | A | NMS4-44-329 | 0.00 | 0.00 |
| Glyma.17G107600 | G | A | N7103 | 0.80 | 0.81 |
| Glyma.17G140600 | T | C | N7103 | 0.80 | 0.75 |
| Glyma.17G202500 | T | C | N7103 | 0.00 | 0.00 |
| Glyma.17G205900 | T | C | NMS4-44-329 | 0.03 | 0.01 |
| Glyma.17G212200 | G | A | NMS4-44-329 | 0.97 | 0.98 |
| Glyma.17G214100 | T | A | NMS4-44-329 | 0.00 | 0.00 |
| Glyma.18G003100 | T | A | NMS4-44-329 | 0.03 | 0.01 |
| Glyma.18G003100 | C | A | NMS4-44-329 | 0.09 | 0.12 |
| Glyma.18G039000 | C | T | NMS4-44-329 | 0.80 | 0.79 |
| Glyma.18G039500 | C | G | NMS4-44-329 | 0.74 | 0.73 |
| Glyma.18G042900 | C | G | NMS4-44-329 | 0.78 | 0.82 |
| Glyma.18G043100 | G | T | NMS4-44-329 | 0.85 | 0.76 |
| Glyma.18G061500 | A | C | NMS4-44-329 | 0.76 | 0.75 |
| Glyma.18G096300 | T | C | N7103 | 0.76 | 0.77 |
| Glyma.18G112500 | A | G | NMS4-44-329 | 0.00 | 0.00 |
| Glyma.18G112500 | G | A | NMS4-44-329 | 0.00 | 0.00 |
| Glyma.18G112500 | T | C | NMS4-44-329 | 0.00 | 0.00 |
| Glyma.18G116400 | T | C | NMS4-44-329 | 0.73 | 0.77 |
| Glyma.18G141500 | T | G | NMS4-44-329 | 0.00 | 0.00 |
| Glyma.18G141500 | T | C | NMS4-44-329 | 0.00 | 0.00 |
| Glyma.19G121600 | T | G | N7103 | 0.78 | 0.76 |
| Glyma.19G123800 | G | C | NMS4-44-329 | 0.85 | 0.85 |
| Glyma.19G130800 | A | G | NMS4-44-329 | 0.76 | 0.72 |
| Glyma.19G219600 | C | T | N7103 | 0.77 | 0.75 |
| Glyma.19G246600 | T | C | NMS4-44-329 | 0.84 | 0.77 |
| Glyma.19G248700 | T | C | N7103 | 0.85 | 0.79 |
| Glyma.19G255500 | C | T | N7103 | 0.12 | 0.06 |
| Glyma.19G255500 | T | C | N7103 | 0.20 | 0.06 |
| Glyma.19G255500 | T | C | N7103 | 0.21 | 0.08 |
| Glyma.19G255500 | A | G | N7103 | 0.20 | 0.07 |
| Glyma.19G255800 | T | G | N7103 | 0.78 | 0.75 |
| Glyma.20G012000 | C | T | NMS4-44-329 | 0.28 | 0.18 |
| Glyma.20G012000 | G | A | NMS4-44-329 | 0.18 | 0.16 |
| Glyma.20G012000 | G | C | NMS4-44-329 | 0.22 | 0.17 |
| Glyma.20G012000 | C | T | NMS4-44-329 | 0.15 | 0.09 |
| Glyma.20G138100 | A | G | NMS4-44-329 | 0.00 | 0.02 |
| Glyma.20G138800 | A | G | NMS4-44-329 | 0.25 | 0.16 |
| Glyma.20G138700 | T | C | NMS4-44-329 | 0.80 | 0.78 |
| Glyma.20G207200 | T | C | NMS4-44-329 | 0.87 | 0.85 |
| Glyma.20G246800 | G | A | N7103 | 0.20 | 0.23 |
| Glyma.U021400 | G | A | N7103 | 0.82 | 0.81 |
